# Patients’ reasoning regarding the decision to participate in clinical cancer trials: an interview study

**DOI:** 10.1186/s13063-018-2916-9

**Published:** 2018-09-29

**Authors:** Pia Dellson, Kerstin Nilsson, Helena Jernström, Christina Carlsson

**Affiliations:** 1 Department of Clinical Sciences, Division of Oncology and Pathology, Lund University and Skåne University Hospital, Barngatan 4, 221 85 Lund, Sweden; 2 Institute of Health and Care Sciences, Sahlgrenska Academy, University of Gothenburg, Göteborg, Sweden

**Keywords:** Patient experiences, Decision-making, Informed consent, Clinical cancer trial, Qualitative study, Interviews, Patient information, Communication

## Abstract

**Background:**

Clinical cancer trials are crucial for the implementation of new treatments in the clinical setting, but it is equally crucial that patients are given the opportunity to make a well-informed decision about participation. The inclusion process is complex, including both oral and written information about the trial. The process of patients’ decision-making regarding clinical cancer trials has not yet been sufficiently studied. This interview study aims to explore the process of patients’ reasoning regarding the decision to participate in a clinical cancer trial.

**Methods:**

The study is based on 27 individual face-to-face interviews with patients who had decided to participate in a clinical cancer trial. The interviews were audio-recorded and transcribed verbatim and then analysed using inductive content analysis.

**Results:**

Content analysis revealed 17 subthemes grouped into five themes: (1) an unhesitating decision to participate; (2) a decision based on flimsy grounds and guided by emotion; (3) feeling safe and secure with my decision; (4) faced with a choice versus what choice do I have? and (5) hoping for help while helping others. The decision to participate in a clinical cancer trial was often immediate and guided by emotions, based on a trusting relationship with healthcare personnel rather than on careful reading of written information. Palliative patients, in particular, sometimes had unrealistic beliefs about the effectiveness of the trial treatment.

**Conclusions:**

It is vital that the decision to participate in a clinical cancer trial is preceded by an honest dialogue about possible positive and negative effects of the trial treatments, including other options such as supportive care in the palliative setting. Our findings also raise the questions of how important written information is for the decision-making process and also whether genuine informed consent is possible. To reach a higher degree of informed consent, it is most important that the oral information is given in a thorough and unbiased manner.

## Background

Including cancer patients in clinical trials is crucial in order to accelerate the process of implementing new treatments in the clinical setting. However, it is equally important that the patients are able to understand the prerequisites of a clinical trial and are given the opportunity to make a well-informed decision about participation. Patient information, which is typically oral as well as written, has the major aim of achieving informed consent to guarantee patients’ autonomy and protect patients from potential harm as stated in the Declaration of Helsinki [1]. However, the written information is complex and needs to consider a large number of issues, e.g. the rationale for different treatment principles and regimes, treatment alternatives, planned procedures, potential risks and benefits, time implications, the right to withdraw at any time without explanation and a number of legal issues. The written information can therefore be extensive and difficult to understand [2, 3]. Support, such as decision aids for patients, guidelines for professionals and standard question guides for physicians, may improve patients’ understanding [4–6]. Several studies have indicated that although patients are generally satisfied with the consent process, understanding is suboptimal of, for example, study aims, treatment risks and benefits, randomisation and research aspects [7–9]. A number of factors have been identified that affect patients’ decision-making regarding clinical cancer trials. Two of the most common factors, particularly in palliative settings, are hope for potential treatment benefit, as well as altruistic motives, as reported by Harrop et al. [10]. Another factor is the concept of clinical equipoise. This is the notion that all treatment arms in a trial are equally effective, which is an underlying motive for randomisation. Whether this idea is acceptable to the patient can also influence the decision to participate or not according to a study by Mills et al. [11]. However, the decision-making process in clinical cancer trials has not yet been sufficiently studied and requires further investigation [12, 13].

A review by Bell and Balneaves [14] identified three types of factors influencing cancer patients’ decision-making for participation in clinical trials: personal; social; and system factors. However, how these factors interact and influence patients’ decision-making process is insufficiently studied and understood. More extensive and deeper understanding of the patient reasoning process regarding participation in clinical cancer trials may provide new insights into patients’ views and vulnerability as well as other factors, both internal and external [15]. This understanding will also benefit patients and guide healthcare professionals in the planning and delivery of future treatments. Therefore, the study aim has been to explore patients’ reasoning regarding the decision to participate in a clinical cancer trial. The method used was individual face-to-face interviews with patients who had decided to enter a clinical cancer trial and the data were analysed with inductive content analysis.

## Methods

A qualitative design [16] was used to explore patients’ reasoning on participation in a clinical cancer trial. Since the intention was to understand patient perspectives, the data collection method consisted of open-ended face-to-face interviews [17]. The inductive content analysis method was used to analyse the data since this method allows analysis at varying levels of abstraction and interpretation [18].

### Setting, participants and data collection

The study was carried out at the two oncology clinics at a university hospital in southern Sweden, over a period of three years. The research nurse who enrolled the patient in a clinical cancer trial also presented the letter of information about voluntary participation in our interview study. This letter was handed out after the patient had agreed to participate in the respective clinical cancer trial, at the first or second appointment, i.e. early during the treatment. The patient signed a letter of consent addressed to the interview coordinator (CC) who then arranged a time for the interview. The study was approved by the Ethics Committee of Lund University (346/2007).

The interviews were carried out by two of the authors (PD, CC) and an external interviewer (GG, see “Acknowledgements”). The patient and the interviewer were the only people present in the room. Each patient was assigned a number to ensure confidentiality. Twenty-seven patients (15 women and 12 men; age range 37–76 years; median age 62 years) were included. Eleven patients received curative treatment and 16 patients received palliative treatment. The patients were diagnosed with ten different cancer diseases (prostate cancer, head-neck cancer, malignant melanoma, ovarian cancer, lymphoma, cervical cancer, breast cancer, Ewing sarcoma, lung cancer and gastrointestinal stromal tumour). They participated in one of 17 different clinical trials, with study phases ranging from I to III. The treatments studied included chemotherapy (*n* = 4), immunotherapy (*n* = 12) and radiotherapy (*n* = 1). The various characteristics of the patients included were considered to be representative of the patient panorama at the clinics.

Before the interviews, the patients were given the opportunity to ask questions. The interview was initiated with the phrase ‘I would like you to describe your reasoning regarding the decision to participate in the clinical cancer trial’. Follow-up questions such as ‘can you tell me more…’ were used. The interviewers had also identified three areas to explore in more detail, prompting the patients to talk about their feelings regarding their decision, about the role of other people who were important in their decision-making and about the study information given to them, particularly concerning the expected side effects. The interviews were audio-recorded, lasted 20–60 min and were transcribed verbatim.

### Data analysis

The transcribed texts were analysed using inductive content analysis [18, 19]. Three authors (PD, KN, CC) read and re-read the transcribed interview texts to become familiar with the data. The transcribed interviews were then imported into the programme NVivo™ 10 (a software for qualitative analysis by QSR International Pty Ltd). NVivo™ was used to organise data during the analysis process. The iterative analysis started with identifying words, statements and sentences, i.e. meaning units, in the interview texts that were related to the study aim. These meaning units were coded according to content. The meaning units with their codes were then re-read and discussed by the authors and grouped into subthemes related to their content. This process led to increased understanding of the actual phenomenon and as the work proceeded, the content of each subtheme was expanded or condensed. In the next analysis step, the authors discussed consistency and finally five themes were identified and formulated that unified the content in the subthemes.

## Results

The themes and subthemes are presented in Table 1. The subthemes that make up the themes are indicated in italics after each theme’s definition. Each subtheme is then described and illustrated below with quotes from different interview patients.

**Table 1:** Overview of the results

Themes	Subthemes
1. An unhesitating decision to participate	- an obvious decision - an immediate decision - an easy decision
2. A decision based on flimsy grounds and guided by emotion	- skimming through the text - registering fragments of information - trusting gut feeling
3. Feeling safe and secure with my decision	- trusting health professionals - feeling safe and secure - extra check-ups
4. Faced with a choice versus what choice do I have?	- keep on living - not having a choice - a chance of recovering health
5. Hoping for help while helping others	- hope of overcoming the cancer - nothing to lose - for my own good - helping others - contributing to research

### Theme 1: an unhesitating decision to participate

Patients talked about their *obvious decision* or *immediate decision* when offered participation in a clinical trial. When they were informed of a clinical trial that matched their form of cancer, they thought in most cases that it was a matter of course to participate, as they wanted to receive the best treatment and considered the trial to be an opportunity to achieve that. However, it was also a matter of course to participate since patients wished to contribute to the further development of new cancer treatments. These initial considerations were not altered by the information given later regarding the clinical trials:

It was quite obvious what I would choose… it didn’t cross my mind not to do what he said [the doctor]… and then I looked at the papers and all the side effects and so on, but that doesn’t really bother me. (Patient 5)

Some patients had already been informed by their referring physician about the possibility of participating in a clinical trial, which gave them time to consider the question beforehand, possibly contributing to their rapid decision. The decision was often made before they informed their next of kin about the offer to participate in a clinical trial. The dialogue with their next of kin had the function of support and to strengthen or confirm a decision already made. However, some patients, although they were few, needed a night’s sleep before making the decision. Many patients immediately announced their decision, but even if they did so they had an exhaustive dialogue with the physician treating them or the researcher about the purpose of the trial and its side effects. Despite information about the risk of side effects, patients valued the chance of receiving potentially effective treatment for their cancer higher than the risk of side effects. Patients were satisfied with their immediate decision and were not anxious about it. When stating their reasons for making an immediate decision, some of the patients referred to their basic attitude towards research, as well as to the opportunity of helping others to recover:

[I hope] it will bring research forward. That they [the researchers] will come up with, that there may be something else, I don’t know, when it comes to medicines or whatever. So that anything at all, that can help, so that people will feel better when they’re affected by this [cancer]. (Patient 14)

Patients considered it an *easy decision* to consent to participation in a clinical trial. The perceived benefits of participation contributed to the feeling that the decision was easy to make. Knowing they were able to withdraw from the clinical trial at any time made the decision to participate easier, for example, if unexpected side effects should occur. Patients given the opportunity to receive an additional treatment with another mode of action, such as an antibody in addition to chemotherapy, considered this treatment to be a sensible complement to the standard treatment:

I would maybe not have participated in just any study, but this, well, I believe in the idea, it seems sensible to me, so it was a really easy decision. (Patient 22)

### Theme 2: a decision based on flimsy grounds and guided by emotion

Before patients decided to participate in a clinical trial, they received both oral and written information. However, patients did not give the written information their full attention and did not read it verbatim, only *skimming through the text*. Some read only the headings and ignored the parts that appeared less interesting and others partook of the written information only because they felt obliged to. Whether they felt an interest or were able to understand everything was of less importance to them. Instead they followed their initial feeling that this was something good of which they wanted to be a part. These patients were not interested in exhaustive information, being content with only an overview, and stating they were persons who did not ask many questions. They were also informed about potential side effects of the treatments included in the study. They had considered the risk of side effects before arriving at their decision to participate, but this information seems not to have influenced their decision:

[I had] skimmed through it [the written information], and then after that I had no doubts, like. Of course, you can get side effects, you can get them from all medicines. (Patient 18)

Patients did not experience the question of participating in a clinical trial as traumatic; it was the disease itself that was traumatic. They felt that participating in a study was positive as it enhanced their psychological wellbeing and sense of confidence. Patients reported that they were so occupied by thoughts and feelings related to their cancer and treatment that they were unable to take in the information given, irrespective of whether it was oral or written. Therefore, they only *registered fragments of information* before making a decision. Patients were guided by their feeling that it was the right thing to do and *trusted their gut feeling*, even if they sometimes partook of more information later on to validate their decision:

My gut feeling told me directly that I should join, but I didn’t say either yes or no just then, only took the papers home with me. And when I had read the papers I decided that I would participate. (Patient 10)

### Theme 3: feeling safe and secure with my decision

Patients *trusted healthcare professionals* at the University Hospital and this trust influenced their decision to participate in the clinical trial. The University Hospital was expected to have special resources and the healthcare professionals were considered well educated. At a moment when patients themselves felt irresolute, they trusted their physicians’ knowledge; they were the experts. Research nurses were also apprehended as providing special attention, since patients perceived them as their own nurse:

You trust an authority, don’t you? And I kind of believe that the doctors here, who are like specialised in this sphere of diseases, of course you trust them. Who else would you trust? (Patient 5)

The patients’ trusting relationship with the healthcare professionals made them *feel safe and secure* about the decision they had made. In addition to trusting the healthcare professionals, patients talked about the trust they had in the research community and how they were convinced that the clinical trials were based on certain assumptions about the positive effects of the drugs being tested. The patients understood that they would receive extra check-ups in addition to the regular ones and often with a longer follow-up time. These factors contributed to creating a sense of security, irrespective of whether the patients were allocated to the control or treatment arm of a study:

The best part was that even if you didn’t get to have this [new medicine], you’re still in the control and get kind of a longer follow-up. It felt like you got a little extra security somehow. (Patient 4)

*Being checked up* gave patients hope that any future recurrence of the cancer would be detected earlier than otherwise. In addition to receiving more numerous and regular check-ups, patients were of the opinion that participating in a clinical trial would imply receiving better healthcare.

### Theme 4: faced with a choice versus what choice do I have?

For some patients, the decision to participate was strengthened by the knowledge that all other treatment options had been exhausted. These patients were staged with advanced cancers, where the standard treatment had not had the desired effect. They stated that they had been asked to participate knowing that their current treatment was ineffective. They therefore reasoned that the situation could not become worse by participation in the trial. In this situation patients felt they did not have any choice. The desire *to keep on living* influenced their decision to participate. Patients expressed feeling compelled to do everything in their power and to seize any opportunity for recovery:

I feel I have no choice. I do want to get well, if that’s possible, and then you have to catch at all the straws you can find. (Patient 7)

Not only lack of treatment effect gave patients a feeling of *not having a choice*. Even if physicians gave the impression there was a democratic choice whether to participate, patients felt they had to rely on the physicians’ expert knowledge. Several patients considered it not necessary to have knowledge of their own prior to a decision to participate. They trusted the physician as having sound judgement:

I don’t have much choice, do I? I have to rely on the doctor’s expertise and trust in his knowledge. I don’t have the knowledge to say ‘no, I don’t think I need to take part in that study’. (Patient 11)

Independently of why patients experienced not having a choice to participate or not, they felt that participation in a clinical trial might offer *a chance to recover health* and rid themselves of their cancer. Patients hoped for recovery or at least a chance to prolong life. Though not knowing for certain whether they would receive the new treatment, participating in the clinical trial gave them a possibility to receive a new treatment otherwise not available:

I know that this study of a supplementary [drug], it’s a kind of drug that inhibits the development of new blood vessels to the tumour and it is known to be very effective. And you can’t ask to have this treatment any other way. The only chance to get it is if you participate in the study. (Patient 26)

### Theme 5: hoping for help while helping others

The patients’ reasoning regarding participation in a clinical trial included the hope of overcoming their cancer, in part for their own sake and partly for future patients. These aspects were intertwined. Most patients began by talking about the hope of recovery for their own part, but some began by talking about possible future benefit to others. Participation in a clinical trial was seen as a positive offer and when given that opportunity they felt they wanted to give it a try. While talking about *hope of overcoming the cancer*, patients at a palliative stage sometimes talked simultaneously about the advanced stage of their cancer and how serious their situation was considered to be. Those with advanced disease said they had *nothing to lose* by joining the study; all attempts were worth trying:

I intend to try all means available to get better, or at least not to get any worse than I am right now, so I feel that I have nothing to lose by joining a study. (Patient 1)

Patients who had received various treatments previously without any effect on the cancer or who had even experienced deterioration stated that they hoped to receive a more effective treatment by participating in the clinical trial. In this situation, they put themselves and their survival first and did not worry about potential side effects. They hoped that the clinical trial would *be good for them*:

It is a deadly disease so number one, priority one, well that’s to survive. Then if one suffers any side effects, for me that doesn’t feel so important. (Patient 17)

In contrast, patients who said that the clinical trial was perhaps a futile last straw could put other people first and themselves second in their statements. They were aware that they would possibly not have any benefit from participating in the clinical trial but were motivated to participate by knowing they could be *helping others* in the future. They wanted to contribute to developing new treatment strategies by participating. They referred to previous patients taking part in earlier studies and their contribution to current treatments, which motivated them to participate:

It does say in the papers that this isn’t anything that you yourself will benefit directly from, but that it’s useful for future patients, and then I thought that (…) if I can be of help it’s a good thing, and since they don’t know anything one might as well be a bit of a guinea pig, also since I can drop out whenever I want to. (Patient 2)

Patients suffering from a genetic cancer talked about the threat to their children and other relatives, who were at risk of developing the same cancer, and this affected their decision. They wanted to participate to further new treatment solutions.

Patients also talked about contributing to research in more general terms. *Contributing to research* was the primary cause for some patients to accept participation. They considered this to be important and felt satisfaction in contributing to bringing research forward. These statements were also related to patients’ interest in research and above all the progress of research, in discovery and improvement, and they were also interested in the final result of these studies:

And then I actually thought that this is an advanced research project where you have an almost finished medication that you are just testing against sugar pills. So I thought ‘this is my contribution to research’. (Patient 24)

## Discussion

The study identified 17 subthemes grouped into five major themes. The most striking finding was that many of our informants described the decision to participate as instant, taken even before they had been given full information about the clinical trial in question. The decision was guided more by emotion than conscious deliberation. The patients stated that the decision to participate was obvious, immediate and easy. It was not guided by the written or oral information on the trial, which they only skimmed through or registered fragments of, but rather based on a positive gut feeling.

This immediateness of patients’ decision-making process has, to our knowledge, rarely been described before [20] and it can be understood through theories in cognitive psychology. Extensive work has been done regarding theories of dual processing in decision-making, where fast intuitive and emotion-driven System 1 processes are contrasted with the slow analytic procedures of System 2 [21, 22]. It is assumed that the fast System 1 processes cue default intuitive judgements, that in some cases are later endorsed by the analytic System 2. This is in accordance with our findings, where the patients described an immediate decision to participate, based on hopeful and positive feelings towards the physician and research in general. This fast, emotional process was sometimes followed by a slower cognitive process. Here, they examined the oral and written clinical trial information more closely and discussed their decision with others to confirm it. However, some did not find this slower process necessary but were satisfied with their immediate decision. This fact points to the importance of not regarding the decision-making process as solely, or even primarily, a cognitive process. A study by Yang et al. confirms the importance of emotions in cancer patients’ decision-making, presenting data that emotions play a significantly greater role for them compared to a representative national sample [23]. Taken together, this suggests that written information may be less important for the decision process than is generally assumed.

In some cases, the referring physician had taken up the issue of clinical trials before the patient arrived at the oncology department; this may have contributed to a faster decision-making process there, as the patient had time to consider the question beforehand. Furthermore, epidemiological studies have shown that pre-notification significantly increased participation in clinical trials [24]. This is in line with other research [25] and points to the importance of regarding informed consent not as a single event but rather as a multistage process.

Another interesting finding is that the patients attached great importance to the relational aspects of the decision-making process, referring to the fact that they trusted the physicians, the research nurses and researchers. This trust made them feel safe and secure with their decision to participate in the clinical trial, which is in line with previous studies [26, 27]. Another important aspect was that trial participation would result in closer follow-up and more direct access to care, through the research nurse in particular. Many of our patients were also convinced that the clinical trials were based on certain assumptions about the positive effects of the drugs being tested and, despite information about the risk of side effects, patients valued the chance of receiving effective treatment for their cancer so highly that they accepted the risk of side effects. This finding is in accordance with Madsen et al. [28] who found that trial participants tended to concentrate on possible differences in treatment effects and often considered the experimental treatment option to be superior. This misconception is an important issue to address in order to achieve informed consent based on a correct understanding of the trial design.

Some patients described not actually having the choice to decline, either because they felt they had to rely on the physician’s knowledge, which they considered more expert than their own, or due to the fact that participating in the trial was the only possibility to access further treatment for their form of cancer. These patients were often in a palliative situation. Under a serious life threat, there may be even more difficulties in cognitively processing information about a clinical trial. Furthermore, some patients had an unrealistic belief that the treatment was curative due to not understanding that the cancer situation was palliative. Again, this raises the question as to whether truly informed consent actually exists under these circumstances [29]. Moreover, many physicians find it difficult to bring up the issue of palliative care as a treatment option, due to fear of taking away the patient’s hope, thus tending to avoid the subject altogether [30]. It is therefore vital that the decision to participate in a clinical trial is preceded by an honest discussion about the possible positive and negative effects, including other existing treatment options where one of the options described should be palliative or best supportive care. This approach is also suggested by Godskesen [31] and is of great importance since supportive care may actually lead to increased life expectancy with better symptom control than a continued tumour-directed treatment, as shown in a lung cancer study by Temel et al. [32].

Our most expected finding was the theme ‘Hoping for help while helping others’, which is in accordance with other studies, where these aspects were the main reasons for cancer patients to participate in a clinical trial [10, 33–35]. The patients were grateful to previous patients who had contributed to the current treatments and wanted to make the same effort for future patients. They considered themselves part of a context in which different contributors cooperate for the common good. This reasoning can be considered a kind of coping strategy aimed at making a difficult situation more meaningful, as described earlier by Godskesen et al. [36].

### Study trustworthiness

The trustworthiness of the study results can be discussed in relation to credibility, confirmability, dependability and transferability [18, 37–39]. The credibility of the study was strengthened by the use of open-ended interview questions, which made it possible for the patients to speak freely about their reasoning regarding participation in a clinical cancer trial. An interview guide was used to ensure that each interview covered topics that were considered central. The sample size of 27 patients was deemed sufficient to reach understanding of the phenomenon in question.

The three authors performing the analysis have different backgrounds and experiences. The first author is a consultant physician in oncology and psychiatry. The second is a specialist nurse with a PhD in the field of patient perspective and involvement and the third is a registered nurse and professor in healthcare pedagogics. These differences have been valuable during the process of analysis to reinforce confirmability, as they have facilitated the reflexivity of the analysers concerning preconceptions and interpretations during the analytic process. The analysers’ different professional backgrounds, knowledge and experience of meeting patients in clinical settings have contributed to a richer and more developed understanding and interpretation of the patients’ reasoning.

The dependability of the study has been supported by the accurate and detailed description of the data collection and analysis. The open-ended interviews have contributed to obtaining varied and rich descriptions of the patients’ reasoning. The patients’ quotes illustrating the themes and subthemes strengthen the consistency of the results.

### Limitations and strengths

The most important limitation is that we only included patients who had agreed to participate in a clinical trial, not those who had declined participation. Such selection of informants may only cover certain aspects of the decision process and hence give an incomplete picture. For example, our finding that few of the patients were concerned about the side effects of the trial treatments may in part be explained by the fact that this is a main reason for patients to decline participation [28]. Consequently, our study sample would rarely include patients with these opinions. Furthermore, the concept of clinical equipoise was referred to in accepting terms, as helping out by being ‘a bit of a guinea pig’ when the experts do not know which treatment is best. This is in accordance with Mills et al., who found that patients accepting the idea of clinical equipoise tend to consent to randomised trials [11]. However, the immediate decision process described by our patients may also be present in patients declining to participate and would be interesting to address in another study.

Our reason not to include any declining patients was based on an ethical consideration of not wanting seemingly to call their decision into question through our examination of the decision process. This may have been too cautious, and the disadvantage of not including the decliners is that they may have provided valuable insights into the obstacles to participation in clinical trials that patients experience, which in turn may have contributed to the improvement of inclusion procedures in the future. According to these findings, it is very important to carry out a further study on the reasoning of patients who declined participation.

The study has several strengths. The patients were interviewed soon after their decision to participate, which decreased the risk of memory errors. Moreover, this proximity in time ensured that the views of patients in a late palliative stage were also captured. The patients included represented many different aspects of cancer patients and trials. The patients were men and women of different ages. They had different cancer diagnoses at different stages, both curative and palliative, and participated in many different clinical trials ranging from phase I to III, testing a range of different treatment modalities. This diversity is an important aspect of qualitative studies with an explorative intent.

The findings in this study, as in other qualitative studies, do not claim to be generalizable. However, given the above, the results of this study should be transferable to similar contexts in other western countries.

## Conclusions

This study suggests that the decision to participate in clinical cancer trials is often immediate and guided by emotions, based on a trusting relationship with healthcare personnel rather than on careful reading of written information. It is vital that the decision to participate is preceded by an honest dialogue about the possible positive and negative effects, including other options such as supportive care in the palliative setting. Our findings may even raise the question of whether genuine informed consent is possible and also of how important written information is for the decision-making process. In conclusion, to reach a higher degree of informed consent, it is of great importance that oral information is given in a thorough and unbiased manner.
